# Behavioral variant frontotemporal dementia patients do not succumb to the Allais paradox

**DOI:** 10.3389/fnins.2014.00287

**Published:** 2014-09-10

**Authors:** Maxime Bertoux, Florian Cova, Mathias Pessiglione, Ming Hsu, Bruno Dubois, Sacha Bourgeois-Gironde

**Affiliations:** ^1^Institut Jean Nicod, Ecole Normale SupérieureParis, France; ^2^Swiss Centre in Affective Sciences, University of GenevaGeneva, Switzerland; ^3^Institut du Cerveau et de la Moelle Epinière, INSERM UMRS 975, Hôpital Pitié-SalpêtrièreParis, France; ^4^Neuroeconomics Laboratory, Haas School of Business, University of California, BerkeleyBerkeley, CA, USA; ^5^LEMMA, Université Panthéon-AssasParis, France

**Keywords:** Allais paradox, anticipated regret, emotions, rationality, frontotemporal dementia

## Abstract

The Allais Paradox represents one of the earliest empirical challenges to normative models of decision-making, and suggests that choices in one part of a gamble may depend on the possible outcome in another, independent, part of the gamble—a violation of the so-called “independence axiom.” To account for Allaisian behavior, one well-known class of models propose that individuals' choices are influenced not only by possible outcomes resulting from one's choices, but also the anticipation of regret for foregone options. Here we test the regret hypothesis using a population of patients with behavioral variant frontotemporal dementia (bvFTD), a clinical population known to present ventromedial prefrontal cortex dysfunctions and associated with impaired regret processing in previous studies of decision-making. Compared to matched controls and Alzheimer's disease (AD) patients, we found a striking diminution of Allaisian behavior among bvFTD patients. These results are consistent with the regret hypothesis and furthermore suggest a crucial role for prefrontal regions in choices that typically stands in contradiction with a basic axiom of rational decision-making.

## Introduction

In the Allais paradox, an individual is asked to make two decisions corresponding to two sets of prospects. The first decision involves a choice between the following Lotteries A and B.

– Lottery A: The player receives 500,000 € with 100% of certainty.
– Lottery B: The player receives 500,000 € with a chance of 89% or 2,500,000 € with a chance of 10% and gets nothing with a chance of 1%.

Then a second decision is made regarding Lotteries C and D:

– Lottery C: The player gets 500,000 € with a chance of 11% and gets nothing with a chance of 89%.
– Lottery D: The player gets 2,500,000 € with a chance of 10% and nothing with a chance of 90%.

Behaviorally, there is substantial evidence that most individuals prefer A over B then D over C. These, however, are choices that violate standard expected utility theory. If one computes expected payoffs for the above lotteries, it is clear that:

– E(A) = (0.89 + 0.10 + 0.01) × 500,000 = 500,000;
– E(B) = 0.10 × 2,500,000 + 0.89 × 500,000 = 695,000;
– E(C) = (0.10 + 0.01) × 500,000 = 55,000;
– E(D) = 0.10 × 2,500,000 = 250,000.

Thus, a subject is said to exhibit a preference reversal when they move from the lottery [A, B] to the lottery [C, D]. More precisely, they violate a basic assumption of the well-orderliness of preferences and challenge the idea that there exists a well-behaved utility function underlying choice behavior.

In particular, the choice pattern observed with respect to the above Allais choices presents a violation of Savage's sure thing principle or, alternatively, as a violation of the independence axiom central to subjective expected utility theory (Savage, 1954). This axiom states that if *p* is preferred over *p*′, this preference continues to hold up to any linear transformation such that α*p* + (1 − α)*p*″ > α*p*′ + (1 − α)*p*″. In simpler terms, the sure thing principle states that when an outcome is irrelevant, it should not be taken into account in decision-making. As put by Allais in retrospect (Allais, 1953), this principle may be incompatible with the preference for security in the neighborhood of certainty, which is reflected by the elimination of all strategies implying a non-negligible probability of ruin (so B is generally discarded in favor of A).

Allais points here to a psychological process underlying the violation of independence. The certainty or quasi-certainty effect (Kahneman and Tversky, 1979) is indeed one of the prevailing explanations of the Allais paradox. More precisely, it states that when among several options a sure or quasi-certain one is presented, the choice is biased toward that option in spite of a consequent violation of utility maximization or of preferences consistency. Another explanation has been but in terms of regret-theory (Loomes and Sugden, 1982, 1987). The idea is that, when making decisions, individuals take into account not only the consequences they might experience as results of the action chosen, but also how each consequence compares with what they would have experienced under the same state of the world, had they chosen differently. Their satisfaction then undergoes an increment or a decrement according to how much regret or rejoicing they anticipate they will experience when they are in a position to retrospectively compare between what they get and what they could have gotten. These two explanations are not rival and exclusive, in the sense that they jointly point to an effect, possibly of an elaborate affective and cognitive nature such as the anticipation of regret, of sure gains on deflecting people from strictly utility maximizing choices.

Unlike basic emotions such as fear or happiness, regret is a cognitively-based emotion, stemming from the individual feeling of responsibility with respect to the outcome of one's own choice (Canessa et al., 2009). Studies of the neural bases of regret tend to show a ventromedial prefrontal cortex (VMPFC) involvement through the use of different paradigms and methodologies (for a review, see Sommer et al., 2009). Using a decision-making task in which choices between two risky monetary gambles were required, Camille et al. (2004) investigated whether the presence of the feedback about the potential outcome of the unchosen option in a binary choice could lead to a difference in the emotional appraisal of the actual outcome of the chosen option and whether this feedback influenced the choice strategy for subsequent decisions across their experiment.

The authors observed that unlike controls, patients with VMPFC lesions were not influenced by the outcome of the foregone option and reported no regret when learning they could have obtained a better outcome. Using the same paradigm in a functional imaging study on healthy controls, Coricelli et al. (2005) showed that the degree of regret covaried with activity within the VMPFC as well as in the dorsal anterior cingulate. Consistently, the VMPFC activation was reported during the specific experience of regret (by contrast with simple “disappointment” about what you get in a situation when do not in fact compare with what you could have gotten) during a decision-related loss task (Chua et al., 2009). VMPFC again, as well as putamen, have furthermore been linked, in an electrophysiological study, to the so-called “near-miss effect” in gambling outcomes (Qi et al., 2011). While the decision-making process depends on multiple factors such as attitude toward risk, context, or outcome representation, it seems clear that regret plays an important role in normal rewarded decision-making and that VMPFC acts as a key node in the normal processing of this emotion (Shiv et al., 2005; Fellows and Farah, 2007; Sommer et al., 2009).

Thus, if an affective and cognitive process, such as regret-anticipation, plays a role in accounting for the Allais paradox, it is supported by psychological mechanisms that are in turn encoded by a specific neural structure. In this respect our study is, in its principle, congenial to studies on decision-patient behavior among VMPFC patients such as Shiv et al. (2005), and Fellows and Farah's (2007). In order to test our more particular hypothesis with respect to the Allais paradox, we investigate the extent to which Allaisian behavior is affected in patients suffering from a neurodegenerative disease considered as a prototype of VMPFC dysfunctions: the behavioral variant frontotemporal dementia (bvFTD). bvFTD is characterized by hypometabolism and atrophy in frontal and polar temporal lobes (Schroeter et al., 2008; Seeley et al., 2008; Agosta et al., 2012), particularly in the medial and ventro-medial parts of the prefrontal cortex (Boccardi et al., 2005; Perry et al., 2006; Schroeter et al., 2007). Given our interest in understanding the involvement of regret in the Allais paradox, bvFTD is a natural choice since (1) it is considered as a model of VMPFC and medial prefrontal cortex (mPFC) dysfunctions (Lu et al., 2006; Zald and Andreotti, 2010) and (2) that emotional processing impairments have been consistently described in this disease (Bertoux et al., 2012, 2013). As a pathological control group, we decided to administrate this Allais task to mild Alzheimer's disease (AD) patients, in which VMPFC and mPFC integrity has been described as spared (Rabinovici et al., 2007; Tranfaglia et al., 2009), as well as emotional processing (Funkiewiez et al., 2012; Bertoux et al., 2013, 2014a). Finally, we contrasted both groups to age and education matched healthy controls.

We studied Allaisian behavior in participants using a set of sequential choices with no feedback and hypothetical payoffs. Thus, unlike a number of other studies, our focus was not whether subjects were going to maximize their own payoffs, but in terms of consistency across series of choices. In particular, whereas payoffs fluctuates across different environments, choice consistency as defined under subjective expected utility theory provides a much more general test of rationality in the sense of guaranteeing utility-maximization in expectation.

In the first phase, participants chose between pairs of investments (lotteries) that were successively presented to each participant. Each investment was described by a list of potential gains (or losses) and the associated probability (for example: 50% chance of earning 750$, 50% chance of earning nothing). Pairs of investments presented were sufficiently contrasted so that the participants were likely to express their choice on the basis of a criterion such as anticipated regret.

In a second phase, participants made similar choices between two pairs of investments, but were also presented with what outcome would have occurred if they had made the other choice, and were asked to rate their satisfaction with the actual outcome. Under the regret hypothesis, we should observe significantly fewer cases of Allaisian patterns and less sensitivity to potential outcomes in bvFTD patients, due to their deficits in emotion processing.

## Materials and methods

### Participants

A total of 14 bvFTD patients and 13 AD patients in the early or moderate stages of the disease, and 12 age and education matched controls were recruited for the study. All bvFTD and AD patients were seen and evaluated at the Memory and Alzheimer Institute of the Pitié-Salpêtrière Hospital in Paris, France. The final diagnosis was established by experts after multidisciplinary clinical synthesis based on neuropsychological, neurological, biological and neuroimaging evidences. bvFTD patients were enrolled according to the revised Lund and Manchester criteria for bvFTD diagnosis (Neary et al., 1998) and fulfilled the new revised diagnostic criteria for “probable bvFTD” (Rascovsky et al., 2011). All patients had prominent changes in personality and social behavior that were established from their caregivers' interviews. AD patients were enrolled according to the revised NINCDS-ADRDA Criteria (Dubois et al., 2007). They presented with a prominent history of episodic memory impairment with temporal and spatial disorientation and had a clinical dementia rating (Morris, 1993) score ≥ 0.5.

All patients underwent a comprehensive neuropsychological examination. Clinical MRI and/or SPECT scans were performed for all patients and showed frontotemporal atrophy or/and hypoperfusion for bvFTD patients and medial temporal lobe atrophy for AD patients. Patients were excluded from the study if they presented any of the following: (1) substantial language production and comprehension deficits suggesting progressive non-fluent aphasia or semantic dementia; (2) co-morbid medical conditions that could interfere with cognitive functioning; (3) vascular lesions validated by MRI or neurological history suggesting vascular dementia, and (4) motor-neuron disease. To improve diagnostic accuracy, all patients had at least an 18-month follow-up in the National Reference Center for Rare Dementias or in the Resource and Research Memory Center of the Pitié-Salpêtrière Hospital, in order to validate the diagnosis according to their clinical evolution. Clinical and demographical data are shown on Table 1, as well as scores in facial emotions recognition test (please see Funkiewiez et al., 2012 for a complete description of this task).

**Table 1:** **Clinical and demographic data for the participants**.

**Groups**	**Sex ratio (F/M)**	**Age**	**Education (years)**	**MMSE (/30)**	**FAB (/18)**	Facial emotions recognition (%)^*^
bvFTD	6/8	66.7 (9.1)	10.9 (3.9)	24.1 (2.3)	12.4 (2)	40% (11.9)
AD	7/6	78.2 (7.5)	11.3 (3.7)	23.7 (3.2)	14.2 (2.4)	87.5% (8.7)
Controls	8/4	68 (7.5)	11.6 (2.9)	28.9 (0.9)	17.4 (0.5)	N.A.

*Mean (Standard deviation). F/M = Female/Male*.

* Norm for this test is 83.2% (7.47) from Bertoux et al. (2013).

Behaviors of patients were compared to those from twelve age and education matched healthy controls. They were either spouses of unrelated patients or recruited via advertisements in the hospital. Controls were included according to the following criteria: (a) Mini Mental State Examination (MMSE—Folstein et al., 1975) score > or = 27/30 and Frontal Assessment Battery (FAB—Dubois et al., 2000) > or = 16/18; (ii) no history of neurological or psychiatric disorders; (iii) no memory complaint or cognitive impairment; (iii) no history of drug abuse.

This study was conducted at the National Reference Center for Rare Dementias, and in the Neurology Department (Pitié-Salpêtrière Hospital). All clinical data were generated during a routine clinical work-up and were extracted for the purpose of this study. According to French legislation, patients were informed and consented that their data might be used in retrospective clinical research studies.

#### Allais paradox task (first phase)

We adapted the Allais paradox to a repeated Allais task. The experiment was run entirely on a computer. Two instructions screens were first shown to participants inviting them to imagine that they had to invest some of their money in stocks and repeatedly make binary choices among these stocks. Then a succession of 40 choices between two possible “investments” was presented.

***Experimental choices***. we implemented 24 choices between two “investments” that were sequentially displayed: one on the left half of the screen and the second on the right half of the screen. Each investment was described by a list of potential gains (or losses) and the associated probability (e.g., “90% chance of earning $15,000 and 10% chance of earning nothing,” henceforth noted: [+$15,000 (90%), $0 (10%)]). Among these experimental choices, 12 were choices between investments in which the participants could gain money (e.g., “[+$10,000 (100%)] OR [+$15,000 (90%), $0 (10%)]”) and 12 choices between investments in which the participants could lose money (e.g., “[−$6000 (90%), $0 (10%)] OR [−$14,000 (45%), $0 (55%)]”). To allow comparison between gain and losses, the list of choices between losses was the same as the list of choices between gains, except that the word “losing” was substituted to the word “earning” (e.g., the choice between gains “[+$10,000 (100%)] OR [+$15,000 (90%), $0 (10%)]” became the choice between losses “[−$10,000 (100%)] OR [−$15,000 (90%), $0 (10%)]”). For each choice, the experimenter made sure that participants were fully aware of the probabilities. After the choice, no feedback was provided about the outcome of the investment. The choices had different properties that are detailed below.

***First property***. High probabilities vs. Low probabilities choices: each experimental choice had a “twin” supposed to be equivalent from the standpoint of the sure thing principle (e.g., the equivalent for “[+$10,000 (100%)] OR [+$15,000 (90%), $0 (10%)]” was “[+$10,000 (10%), $0 (90%)] OR [+$15,000 (9%), $0 (91)]”). Thus, we had a total of 12 pairs of choices between two investments (6 pairs of choices among potential gains and 6 pairs of choices among potential losses). Within each pair of choices, one choice presented high probabilities (e.g., “[+$10,000 (100%)] OR [+$15,000 (90%), $0 (10%)]”), with a potential gain (or loss) in the neighborhood of certainty, and one choice presented low probabilities (e.g., “[+$10,000 (10%), $0 (90%)] OR [+$15,000 (9%), $0 (91)]”), with no potential gain (or loss) in the neighborhood of certainty.

**Figure 1:**
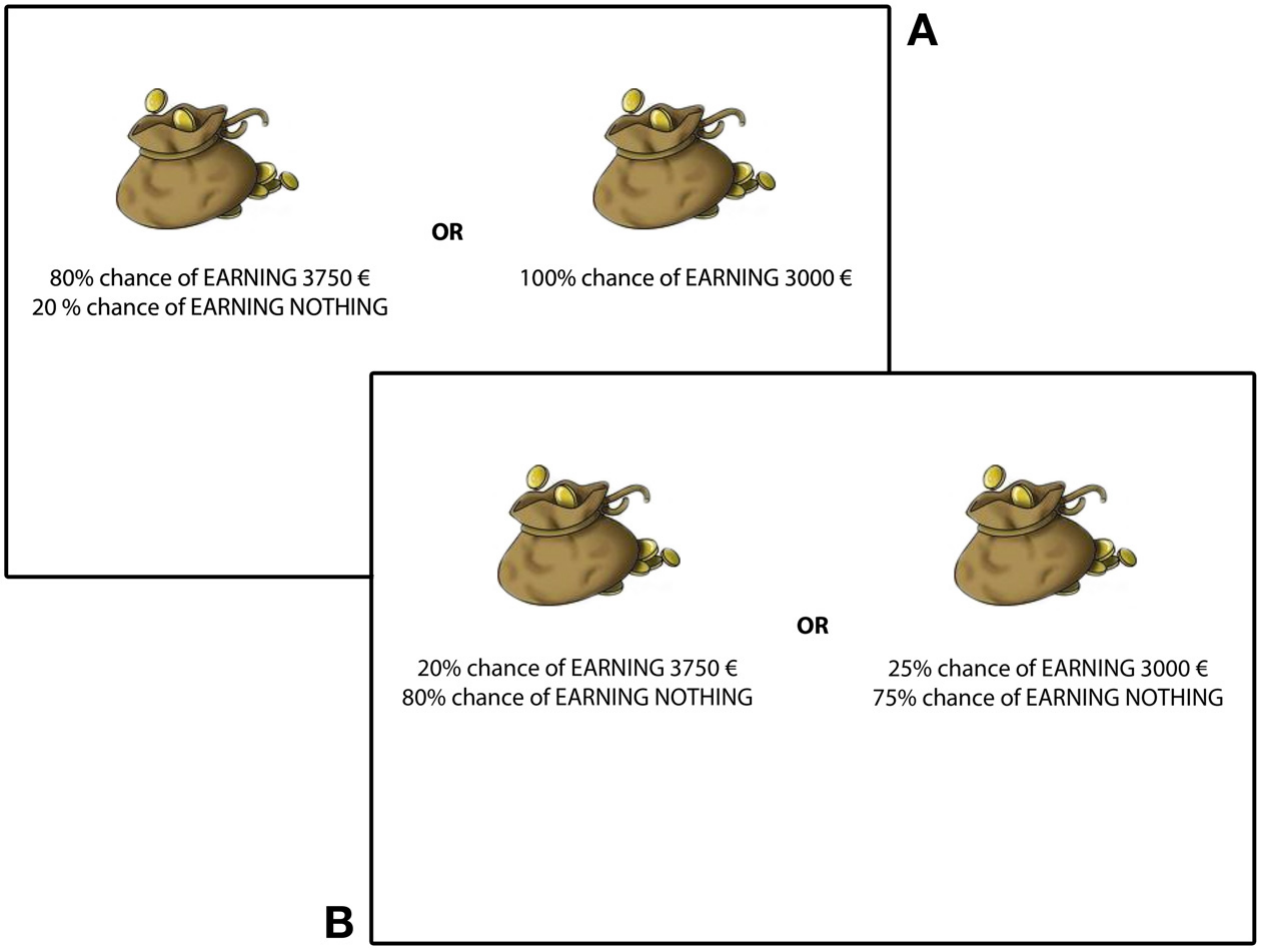
**Two sample pairs of choices between gains.** On top **(A)**, patients have to choose between a risky investment (left) and a secure investment (right) in a high probabilities lottery. Below **(B)**, patients have to choose between risky investment (left) and a secure investment (right) in a low probabilities lottery.

***Second property***. Secure vs. Risky investments: for both kinds of choices (high or low probabilities), one investment presented higher probabilities (of gain or loss) than the other. From now on, we call the secure investment the investment with higher probabilities and the risky investment the investment with lower probabilities (see Figure 1). (For example, in the high probabilities choice “[+$10,000 (100%)] OR [+$15,000 (90%), $0 (10%)],” [+$10,000 (100%)] is the secure investment and [+$15,000 (90%), $0 (10%)] is the risky investment. Similarly, in the low probabilities choice “[+$10,000 (10%), $0 (90%)] OR [+$15,000 (9%), $0 (91)],” [+$10,000 (10%), $0 (90%)] is the secure investment, while [+$15,000 (9%), $0 (91)] is the risky investment.) For all choices, the expected gain (or loss) was higher for the risky investment. For half of the choices, the secure investment had a lower expected utility than the risky investment, while expected utilities were matched for the other half.

***Allaisian scores***. To quantify Allasian behavior, we compared choices in pairs of trials that are equivalent according to the sure thing principle, with one choice featuring a potential gain (or loss) in the neighborhood of certainty (the choice between *high probabilities*) and the other at distance from certainty (the choice between *low probabilities*). This allows us to define Allaisian behavior in an operational way. First, for choices among *gains*, Allaisian behavior consists in choosing more frequently the *secure investment* in the *high probabilities* choices than in the *low probabilities* choices. Second, for choices among *losses*, Allaisian behavior consists in choosing more frequently the *risky investment* in the *high probabilities* choices than in the *low probabilities* choices. This behavioral pattern then corresponds to the modal choice [A, D] in the original matrix presented above. We characterize Allaisian behavior by the occurrence of such a choice pattern in our task.

This definition of Allaisian behavior led us to attribute to each participant two Allaisian score: one for *gains* and one for *losses.* For *gains*, Allaisian score was calculated by subtracting the number of risky investment in high probability choices to the number of risky investment in low probability choices (thus, the score had a potential range from −6 to 6). For *losses*, Allaisian score was calculated by subtracting the number of risky investments in low probability choices from the number of risky investment in high probability choices (again, the score had a potential range from −6 to 6).

***Control trials***. The first four choices of the task were considered as forming a training phase and 12 others were control choices (6 in gains, 6 in losses), in which the investment with the highest expected utility was designed to be the most attractive, either because one choice presented a much higher potential gain or loss (“[±$100 (90%), $0 (10%)] OR [±$1000 (65%), $0 (35%)]”), or because one choice presented much higher probabilities of gain or loss (“[±$3000 (20%), $0 (80%)] OR [±$2000 (80%), $0 (20%)]”), or both (“[±$750 (50%), $0 (50%)] OR [±$1000 (90%), $0 (10%)]”).

A person exhibiting Allaisian behavior will choose the secure investment among the two “high probabilities” investments, but will choose the risky investment among the two “low probabilities” investments, though both choices are equivalent according to the principle of independence.

### Regret task (second phase)

The regret task was presented in a way similar to the Allais task and participants had to make a series of 18 choices between two investments. In each choice, one investment (the *secure investment*) presented a high probability of small gain and a small probability of high loss (e.g., [+$500 (80%), −$500 (20%)]) and the other investment (the *risky investment*) presented a small probability of a high gain and a high probability of a high loss (e.g., [+$2000 (20%), −$2000 (80%)] “20% chance of earning $2000” and “80% chance of losing $2000”). Choices were designed such as participants would normally tend to prefer the secure investment.

However, announced probabilities were not accurate: both the secure and the risky investments had a 50% chance to lead to announced gain and a 50% chance to lead to announced loss. Thus, participants made their choice without being aware of these true probabilities. After each choice, participants were first announced what they had earned (or lost) and then what they would have earned (or lost) if they had chosen the other investment. Finally, participants had to tell whether they were “happy” with their choice's outcome, by indicating their level of satisfaction on a scale ranging from −4 (“NO”) to 4 (“YES”).

For phase 1 and 2 of the test, to avoid any stereotypical or perseveration answer, patients were invited to choose which choice they want to make and a trained clinician (MB) clicked on the selected choice.

### Hypotheses

Our hypothesis with respect to the Allais task was that bvFTD patients would display less Allaisian behavior than control participants and AD patients, i.e., FTD patients would show a significantly lesser discrepancy between their choices in high probabilities and low probabilities, as measured by Allaisian scores.

Concerning the regret task, our hypothesis was that bvFTD patients would show less regret than control participants and AD patients, i.e., their level of satisfaction with one's actual gain (or loss) would be less sensitive to one's potential gain (or loss) for FTD patients than for control participants and AD patients.

## Results

### Demographics and neuropsychological background data

Demographics of all participants are shown in Table 1. These non-normal variables were tested with Mann-Whitney non-parametric test. The three groups didn't differ on educational level (*p* > 0.1). Age was significantly higher in the Alzheimer group (*K* = 15.59; *p* < 0.01) compared to the two other groups that were not different between each other (*p* > 0.1). MMSE and FAB scores were significantly higher in the control groups compared with the bvFTD patients and the AD patient (respectively *K* = 21.77; *p* < 0.001 and *K* = 23.91; *p* < 0.001). Both patient groups did not differ on the MMSE, but were significantly different on the FAB with bvFTD showing significantly more frontal dysfunction than AD patients (*Z* = −2.10; *p* < 0.05). Finally, bvFTD patients showed an impairment in emotion recognition compared to AD (*Z* = −3.15; *p* < 0.0001) who exhibited normal scores at this test [Controls from Bertoux et al., 2014a, obtained 82.5% of good recognition (7.6)].

### Allais task: allaisian behavior (Figure 2)

First, we compared how Allaisian scores difference across groups and conditions. Given that each participant had two scores (one for gains and one for losses), we applied a 3 (*group of participants:* control, AD, or bvFTD; between-subjects) × 2 (*condition:* gains, or losses; within-subjects) ANOVA to Allaisian scores. We found a main effect of *group of participants* [*F*_(2, 36)_ = 10.5, *p* < 0.001] but no main effect of *condition* [*F*_(2, 36)_ = 0.0, *p* = 0.99] and no interaction effect [*F*_(2, 36)_ = 4.9, *p* = 0.16].

**Figure 2:**
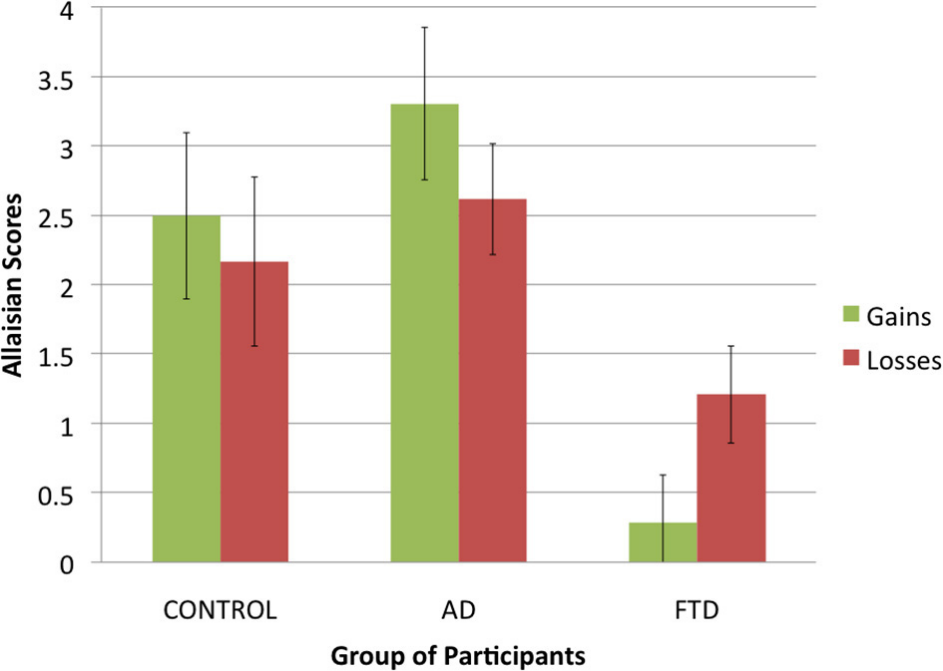
**Allaisian scores in function of group of participants and condition (error bars indicate standard error)**.

**Figure 3:**
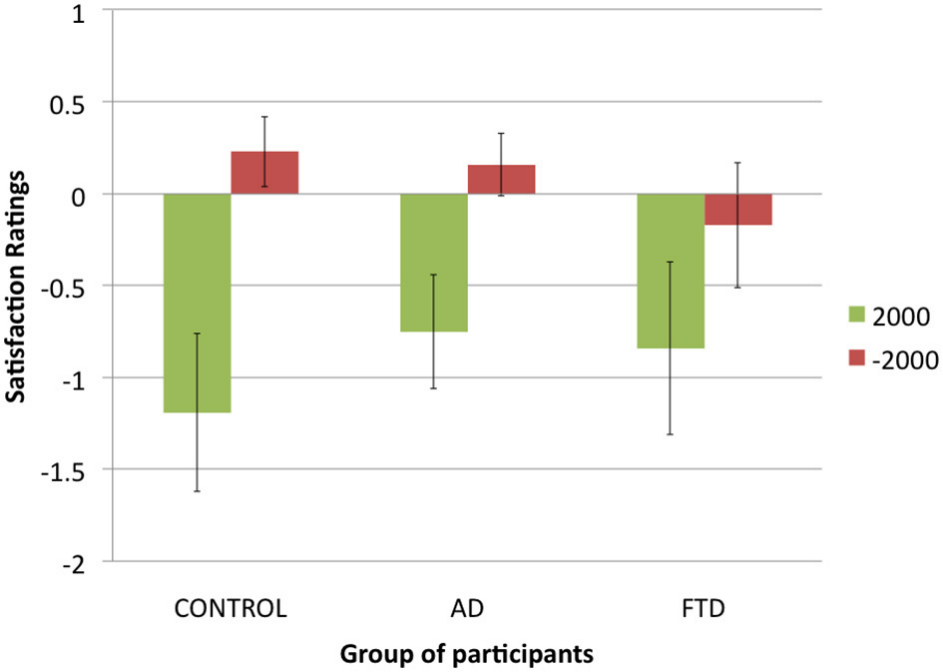
**Number of risky investments in the experimental trials of the Allais task in function of group of participants and condition (error bars indicate standard error)**.

The effect of group was due to bvFTD patients having lower scores than control participants and AD patients (see Figure 3). After summing scores for gains and losses into a single score, we proceeded to pairwise comparison for all three groups using Student *t*-tests. There was no difference between control participants and AD patients (*N* = 25, *t* = −1.1, *df* = 23, *p* = 0.29), but we found a significant difference between both control participants and bvFTD patients (*N* = 26, *t* = 3.6, *df* = 24, *p* < 0.01) and AD patients and bvFTD patients (*N* = 27, *t* = 4.5, *df* = 25, *p* < 0.001).

### Allais task: control trials and risk aversion

An alternative explanation of our results is that bvFTD patients had comprehension and/or motivational differences compared to other participants. For example, more random behavior would lead to lower Allaisian scores. To test for this hypothesis, we analyzed participants' answers to control trials, with the percentage of choices in which each participant chose the investment with the highest expected value as a dependent variable.

For gains, a ANOVA with *group of participants* as a factor and *percentage of maximizing choices* as a dependent variable revealed no significant difference: *F*_(2, 36)_ = 0.2, *p* = 0.82. bvFTD patients made mostly maximizing choices (*M* = 0.75), and no significantly less than control participants (*M* = 0.79) and AD patients (*M* = 0.75).

For losses, a ANOVA with *group of participants* as a factor and *percentage of maximizing choices* as a dependent variable revealed no significant difference: *F*_(2, 36)_ = 2.2, *p* = 0.12. bvFTD patients made mostly maximizing choices (*M* = 0.61), and though they tended to make less than control participants (*M* = 0.74) and AD patients (*M* = 0.81), this difference did not reach statistical significance. Thus, it does not seem that our results can be explained by the random behavior of bvFTD patients.

Another possibility would be that bvFTD patients were less risk averse than control participants and AD patients. To test for this alternative hypothesis, we analyzed the number of risky investments chosen by participants for both experimental choices between gains and experimental choices between losses (see Figure 3 for a presentation of the results). We applied a 3 (*group of participants:* control, AD, or bvFTD; between-subjects) × 2 (*condition:* gains, or losses; within-subjects) ANOVA to the number of risky investments per subject. It revealed a significant main effect of condition [*F*_(1, 36)_ = 7.9, *p* < 0.01], with participants making less risky decision in gains than in losses, which is consistent with previous literature on the subject (Kahneman and Tversky, 1979; Weller et al., 2007). However, there was no main effect of group of participants [*F*_(2, 36)_ = 0.1, *p* = 0.95], suggesting that bvFTD patients were no less risk averse than control participants and AD patients. Finally, we also found a significant trend in the interaction effect [*F*_(2, 36)_ = 2.9, *p* = 0.07], due to the fact that bvFTD patients tended to make no less risky choices in gains than in losses (*M* = 6.43 vs. *M* = 6.21) while there was such a trend in control participants (*M* = 4.5 vs. *M* = 7.67) and AD patients (*M* = 5 vs. *M* = 7.54). This suggests that bvFTD patients were not subject to the framing effect that led control participants and AD patients to treat choices between gains and choices between losses differently.

### Regret task (Figure 4)

In the regret task, we found that bvFTD patients chose the risky investment more often (40% of choices) than control participants (14%) and AD patients (09%). A chi-square test revealed this difference to be statistically significant: χ^2^_(1, *N* = 602)_ = 82.08, *p* < 0.001. This suggests that bvFTD patients were more likely to choose the risky investment in the specific situation when faced with a choice between a risky investment and a quasi-certainty of earning something.

**Figure 4:**
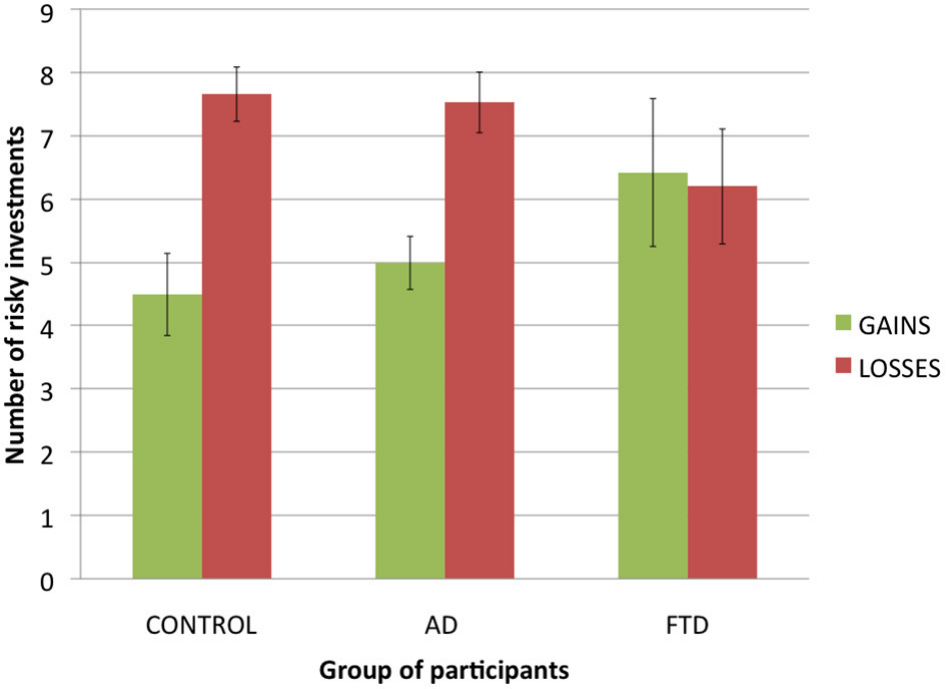
**Satisfaction ratings in function of potential gains (+$2000 or −$2000) for each group of participants (error bars indicate standard error)**.

Because control participants and AD patients chose mainly the secure investment, we discarded from analysis the trials in which participants chose the risky investment, keeping only the trials in which participants chose the secure investment. Then we used a three-factor repeated measures ANOVA with satisfaction ratings as a dependent variable and *actual gain* ($500 or −$500), *potential gain* ($2000 or −$2000) and *group of participants* (control participants, AD patients of FTD patients) as factors. We found a main effect of *actual gain* [*F*_(1, 30)_ = 46.5, *p* < 0.001], with participants giving higher ratings when their actual gain was $500 rather than −$500, a main effect of *potential gain* [*F*_(1, 32)_ = 18.2, *p* < 0.001], with participants giving higher ratings when their potential gain was −$2000 rather than $2000, and no main effect of *group of participants* [*F*_(2, 27)_ = 0.3, *p* = 0.74]. We did not found the expected interaction between potential gain and group of participants [*F*_(2, 32)_ = 0.4, *p* = 0.68], nor an interaction between actual gain and group of participants [*F*_(2, 30)_ = 1.5, *p* = 0.24], suggesting that bvFTD patients did not differ from other participants in how potential and actual gains affected their satisfaction.

However, we found a significant interaction between actual gain and potential gain [*F*_(1, 31)_ = 15.8, *p* < 0.001]: potential gains had a greater impact on participants' ratings when actual gain was −$500 than when actual gain was $500. Finally, we found a significant interaction effect between our three factors [*F*_(2, 31)_ = 4.3, *p* < 0.05]: while control participants and AD patients tended to be more sensitive to potential gains for an actual gain of −$500, bvFTD patients were as sensitive to potential gains for an actual gain of $500. Thus, we did find a difference between bvFTD patients and control participants and AD patients but it was not the one we expected: overall, bvFTD patients did not seem to be less sensitive to potential gains but were differently sensitive to them, taking them into account both for an actual gain and an actual loss, while control participants and AD patients seemed to take potential gains more into account in case of an actual loss.

## Discussion

The aim of this study was to test the extent to which Allaisian behavior is influenced by emotional anticipation. Anticipated regret, in particular, has played a central role in a number of influential models of decision-making seeking to clarify the cognitive and affective processes that underlie Allaisian behavior, and more generally violations of the independence axiom (Bell, 1982; Loomes and Sugden, 1982, 1987; Quiggin, 1994). Based on these works and recent findings from neuroscience, we hypothesized that bvFTD patients would exhibit diminished Allaisian tendencies due to the associated VMPFC dysfunctions and emotional impairments (Bertoux et al., 2012, 2013, 2014a).

Consistent with our prediction, bvFTD was associated with significantly lower Allaisian scores relative to controls and AD patients. Thus, unlike controls and AD patients, who demonstrated a normal violation of the independence axiom and have shown the typical decision inconsistency described by Allais, bvFTD patients were more likely to conform to a basic tenet of rational decision-theoretical behavior. That is, those very same patients who have been previously described to exhibit “irrationality” in terms of an incapacity to maximize their gains in decision-making tasks such as the Iowa Gambling Task (Torralva et al., 2007), are “rational” (albeit in contradiction with the rest of the population) in terms of consistency of choices.

Unlike a number of other gambling tasks, these findings cannot be explained in terms of differences in risk attitude, as Allaisian behavior violates subjective expected utility theory regardless of one's risk attitude. Our findings therefore are in line with previous findings in framing effects and ambiguity aversion that suggest a role for medial prefrontal cortex in detecting and responding to contextual information that result in violation of standard expected utility theory predictions (Hsu et al., 2005; De Martino et al., 2006).

Thus, unlike controls and AD patients, who demonstrated a normal violation of the independence axiom and have shown the typical decision inconsistency described by Allais, bvFTD patients were more likely to conform to a basic tenet of rational decision-theoretical behavior.

Furthermore, we assessed whether bvFTD patients were significantly different in terms of their subjective assessment of *post-hoc* regret. Interestingly, in spite of significantly diminished Allaisian tendencies, we found that bvFTDs appeared to exhibit intact *post-hoc* regret as assessed using self-report. That is, we did not observe significant differences between bvFTD and AD or controls participants, in terms of apparent asymmetric sensitivity to gains and losses. Thus, the results observed in bvFTD about the Allais behavior cannot be straightforwardly linked to bvFTD patients' lack of regret, as we didn't find a significant association between bvFTD patients' sensitivity to potential gains or losses (a behavioral measure of regret) and control participants' sensitivity.

The divergence between Allaisian behavior on the one hand, and regret on the other, raises a number of interesting hypotheses regarding their relationship. First, it is possible that anticipated regret is not a necessary feature of Allaisian behavior. That is, the fact that bvFTD patients exhibited Allaisian behavior while at the same time had intact regret-processing suggests that some other processes underlie Allaisian behavior.

While this study did not provide direct evidence of VMPFC atrophy in bvFTD patients we choose to explore the Allais paradox in bvFTD patients because of its well-known VMPFC dysfunctions in this disease (Boccardi et al., 2005; Perry et al., 2006; Schroeter et al., 2007, 2008; Schroeter, 2012; Bertoux et al., 2013) have led to consider this disease as a good model to study VMPFC functioning (Lu et al., 2006; Zald and Andreotti, 2010) even if the atrophy affects more dorsal median and lateral frontal and temporal areas through the progression of the disease (Agosta et al., 2012). Among the clinical or imaging studies that have investigated the role of VMPFC in the experience of regret, Camille et al. (2004) was the first to make investigate the correlation between this particular frontal area and subjective report of a regret-like emotion in a task liable to arouse it. In that seminal study, subjects needed to choose between two gambles for which the outcome probabilities were indicated. After the choice, depending on the condition, the outcome of the chosen option was uniquely presented only or the two outcomes of the chosen and foregone options were displayed (regret condition). At the end of each trial, subjects were asked to rate their affective state and their skin conductance response was recorded. In the regret condition, by contrast with controls, when patients, the effect of the unobtained outcome was not significant and the emotions they expressed were not modulated by the feedback information concerning the unchosen gamble, leading the authors to conclude that VMPFC patients experienced no regret.

In our study, we investigated how degeneration in brain regions implicated in regret processing impacts Allaisian behavior. Although the observed absence of Allaisian behavior in bvFTD patients suggests a VMPFC involvement, we cannot simply conclude that the behavioral pattern exhibited by our patients in this study can be explained by the fact that they do not experience anticipated regret, given that there is no significant difference in the way our different groups perform with our regret task. The absence of Allaisian behavior in bvFTD was not significantly correlated with the absence of verbal reports of regret on a control task. This divergent result, however, does not dismiss the hypothesis that emotional impairment—whether it is specified in terms of absence of anticipated regret or in other possible less determined terms such as lack of hedonic anticipation or satisfaction associated with future possible outcomes—affects the type of decision made by patients in our Allais task. We must precisely account for a behavioral pattern that shows that the absence of emotional processing associated with VMPFC functions leads to atypical decision behavior in a binary-lotteries task contrasting levels of certainty.

It is possible that bvFTD patients' answers to the satisfaction question in the regret task should not be taken at face value. Indeed, even if bvFTD patients did not experience regret, they can still have an explicit representation of regret and of how potential gains or loss are supposed to impact their satisfaction. A salient possibility, therefore, is that bvFTD patients experienced regret in the regret task but failed to anticipate regret (in the Allais task).

Finally, the atrophic pattern is variable among bvFTD, and we cannot exclude that a representation, if not an attenuated experience of regret, was induced in the regret task in some patients with less pronounced ventro-medial atrophy, given that bvFTD's answers still differed, even though not significantly, from other participants'. Larger samples with quantitative measures of brain degeneration patterns would be necessary to address this important question.

Finally while this study have considered a specific neurodegenerative disease as a model of cognitive impairment to answer to an experimental economy question, our results trend to show that reward-based tests inspired by experimental economic paradigms could be further adapted and employed in clinical setting in order to enhance the differentiation between different conditions, such as bvFTD from AD. Considering the recent evidences of an AD-like episodic memory impairment in bvFTD (Hornberger et al., 2010; Bertoux et al., 2014b) which restrained the clinical distinction between both diseases, these original tests could provide crucial data that could improve the earliness and precision of the diagnosis in the years to come.

Moreover, it is not be reminded that the regret task induced a subjective assessment of satisfaction which was absent from the Allais task, which was purely decisional, not verbal and presenting no feedback. The Allais task was designed in a way in which anticipated regret could play a role in the type of decision-made (leading then to standard Allaisian behavior) whereas the regret task focused on outcomes subjective assessment.

A salient possibility, therefore, is that bvFTD patients experienced regret in the regret task but failed to anticipate regret (in the Allais task). It is also to be emphasized that, like mPFC and VMPFC lesioned patients, bvFTD patients present strong metacognition and emotional deficits that may result in impairments into insights regarding their emotional states (Eslinger et al., 2005; Banks and Weintraub, 2008; Hornberger et al., 2014). Unlike in regret task where participants were repeated ask to focus on their feelings, no such opportunity was present in the Allais task.

An alternative explanation could be put in terms of the effect of risk aversion on decision behavior. Our results in the Allais task showed that bvFTD's behavior could not be explained by a general tendency to take more risks. But we also reported that they nevertheless tended to take more risk in the regret task, where they faced with a choice between a risky investment and a quasi-certainty of earning something. A natural bias toward certain or quasi-certain gains would be lost in bvFTD's patients. This hypothesis is yet to be confirmed by further neuropsychological studies, but this result could consistent with previous studies using decision-making paradigm (Rahman et al., 1999; Torralva et al., 2007) that have shown that bvFTD were risk-takers. Such behavior would also make sense in terms of the role of emotions, associated with risk-aversion or risk-seeking, that has been reported in framing-effect contexts (Kahneman and Frederick, 2007). A framing effect is the fact that two options which are identical in terms of their expected utility give rise nevertheless choice to asymmetrical choice due to the way they are framed in terms of valence. It is admissible to say that the Allais paradox stems from a framing effect at a certain level, given the normative equivalence of the options that nevertheless give rise to systematic divergent choices, and it is quite plausible that elements of risk aversion when contrasting a quasi-certain option with a risky one, are involved in the type of decisions made by our patients. VMPFC activities have been reported to be correlated with risk-averse behavior in framing-effects fMRI experiments (De Martino et al., 2006).

Finally, a third hypothesis could explain results obtained in bvFTD patients while being linked to VMPFC dysfunctions. Indeed, we cannot exclude that the observed pattern in the bvFTD group could be linked to a more general reward-processing impairment due to VMPFC atrophy, as this area has been shown to be a key node of the brain valuation system (Rolls, 2006). An other brain regions involved in reward-processing as well as being atrophied in bvFTD is the ventral striatum (O'Callaghan et al., 2014), and its dysfunctions could have led to a lack of reward anticipation in this patient group during the comparison of choices. While this hypothesis may not be sufficient to entirely explain our results in the Allais task, this whole limbic-system dysfunction could explain why bvFTD patients exhibited a risky behavior in the regret task.

The point of regret-theory is to say that anticipated regret (rather than subjective assessment of *post-hoc* regret) is what explains the violation of the sure thing principle in the problem initially stated by Allais. This is precisely an hypothesis that our results cannot dismiss and even tends to confirm in the light of what is known on the emotional deficits presented by bvFTD patients. bvFTD patients present medial and ventro-medial atrophy that may impair their ability to envision at an experiential level the future consequences of their risky behavior. However, their lack of emotional insight (Eslinger et al., 2005; Banks and Weintraub, 2008; Hornberger et al., 2014) and ability to learn on the basis of past negative experiences (Rahman et al., 1999; Bertoux et al., 2012) might prevent them to fully integrate regret into behavioral optimization patterns. But, at another important level, they also behave in accordance with the normative prescription of economic rationality by actually complying with the fundamental decision-theoretical independence axiom. It is indeed important to note that, unlike other studies in the field of behavioral decision-theory, our focus was not whether subjects were going to maximize their own payoffs. It was differently defined in terms of consistency across series of choices. Consistency has an effect on payoffs in the long run (and actually coincides with utility-maximization), but it constitutes an independent and fundamental dimension of rational choice. The specific question we raised, then, is whether bvFTD patients with VMPFC dysfunctions and healthy controls behave in the same way with respect to making consistent choices. Ironically, the patients were more consistent in that aspect of rationality than their control samples. It means that those very same patients who have been previously described to exhibit “irrationality” in terms of an incapacity to maximize their gains in decision-making tasks such as the Iowa Gambling Task (Torralva et al., 2007), are “rational” (albeit in contradiction with the rest of the population) in terms of consistency of choices. This result suggests interrogations on the descriptive and normative status of principles of rationality and their relation with neuropsychology. In our case we can say that VMPFC atrophy seems to be linked to the absence of deviation from rationality of the type usually entailed by the Allais paradox. By contrast, it shows that a healthy VMPFC is an essential neurobiological structure not to be classically rational.

### Conflict of interest statement

The authors declare that the research was conducted in the absence of any commercial or financial relationships that could be construed as a potential conflict of interest.
